# Serum Lactate for Predicting Cardiac Arrest in the Emergency Department

**DOI:** 10.3390/jcm11020403

**Published:** 2022-01-13

**Authors:** Shu-Hsien Hsu, Po-Hsuan Kao, Tsung-Chien Lu, Chih-Hung Wang, Cheng-Chung Fang, Wei-Tien Chang, Chien-Hua Huang, Chu-Lin Tsai

**Affiliations:** 1 Department of Emergency Medicine, National Taiwan University Hospital, 7 Zhongshan S. Rd, Taipei 100, Taiwan; pedroe929@gmail.com (S.-H.H.); frankkao66@yahoo.com.tw (P.-H.K.); tsungchienlu@gmail.com (T.-C.L.); ogenkidesga@gmail.com (C.-H.W.); conrad@ntu.edu.tw (C.-C.F.); wtchang@ntu.edu.tw (W.-T.C.); chhuang730@gmail.com (C.-H.H.); 2 Department of Emergency Medicine, College of Medicine, National Taiwan University, No.1 Jen Ai Road Section 1, Taipei 100, Taiwan

**Keywords:** emergency department, cardiac arrest, lactate

## Abstract

**Objectives**: Early recognition and prevention of in-hospital cardiac arrest (IHCA) play an increasingly important role in the Chain of Survival. However, clinical tools for predicting IHCA in the emergency department (ED) are scanty. We sought to evaluate the role of serum lactate in predicting ED-based IHCA. **Methods**: Data were retrieved from 733,398 ED visits over a 7-year period in a tertiary medical centre. We selected one ED visit per person and excluded out-of-hospital cardiac arrest, children, or those without lactate measurements. Patient demographics, computerised triage information, and serum lactate levels were extracted. The initial serum lactate levels were grouped into normal (≤2 mmol/L), moderately elevated (2 < lactate ≤ 4), and highly elevated (>4 mmol/L) categories. The primary outcome was ED-based IHCA. **Results**: A total of 17,392 adult patients were included. Of them, 342 (2%) developed IHCA. About 50% of the lactate levels were normal, 30% were moderately elevated, and 20% were highly elevated. In multivariable analysis, the group with highly elevated lactate had an 18-fold increased risk of IHCA (adjusted odds ratio [OR], 18.0; 95% confidence interval [CI], 11.5–28.2), compared with the normal lactate group. In subgroup analysis, the poor lactate-clearance group (<2.5%/h) was associated with a 7.5-fold higher risk of IHCA (adjusted OR, 7.5; 95%CI, 3.7–15.1) compared with the normal clearance group. **Conclusions**: Elevated lactate levels and poor lactate clearance were strongly associated with a higher risk of ED-based IHCA. Clinicians may consider a more liberal sampling of lactate in patients at higher risk of IHCA with follow-up of abnormal levels.

## 1. Introduction

### 1.1. Background

There has been growing research interest in in-hospital cardiac arrest (IHCA) in the emergency department (ED). Patients present to the ED with a wide heterogenicity of potentially life-threatening diseases and are therefore particularly prone to IHCA. Furthermore, the widespread problem of ED overcrowding may increase the likelihood of IHCA due to delays in diagnosis and treatment [1,2,3]. The incidence of IHCA is about 1–6 per 1000 hospital admissions [4,5,6,7], with estimated mortality that ranges from 80–85% [5,8,9,10,11]. IHCA within the ED was reported in approximately 8–11% of the total IHCA population [12,13]. Risk stratification is essential to prevent unidentified deterioration to IHCA while in the ED; however, such a tool for predicting IHCA in the ED is scarce.

### 1.2. Importance

The initial serum lactate level [14] and the lactate clearance [15,16] are useful biomarkers for predicting the prognosis in patients with trauma [17], gastrointestinal bleeding [18], infection [19], pneumonia [20,21], and sepsis [22]. Sepsis is perhaps the most studied ED condition that is related to lactate. Studies have reported that lactate levels function as an inpatient mortality predictor in ED patients with infection and among ED patients with lactate levels ≥4 mmol/L without overt shock [19,23]. Very few lactate studies included a wider spectrum of ED patients. A study reported that higher ED lactate levels were associated with increased in-hospital mortality in patients aged >65 years, irrespective of the presence of infection [24]. Another cohort study of 2272 patients showed that ED lactate was associated with in-hospital mortality among critically ill ED patients [25]. To the best of our knowledge, there have been no ED studies focusing on the relationship between lactate and ED-based IHCA. Thus, the predictive value of lactate level and lactate clearance for IHCA in the ED remains unclear.

### 1.3. Goal of This Investigation

In this study, we sought to evaluate the predictive value of serum lactate, including the initial lactate levels and lactate clearance, for IHCA occurring in the ED.

## 2. Methods

### 2.1. Study Design and Setting

We conducted a retrospective cohort study using data stored within the Integrated Medical Database (iMD) of the National Taiwan University Hospital (NTUH). The iMD serves as a central clinical data repository for all electronic medical records of the healthcare system for one chief hospital and six affiliated hospitals and includes inpatient, outpatient, and ED records. It houses various information, including demographics, diagnosis, treatment, imaging, laboratory, prescription, nursing, billing, and administrative data. The iMD is maintained and updated by dedicated research personnel and has been used in several clinical research studies [26,27].

For the current study, we retrieved de-identified ED data from the chief hospital over a 7-year period between 1 January 2009 and 31 December 2015. The chief NTUH hospital is a tertiary academic medical centre with approximately 2400 beds that receive 100,000 ED visits per year. This study was approved by the Institutional Review Board of the NTUH, which waived the requirement for informed patient consent for this de-identified study.

### 2.2. Study Population

**Figure 1:**
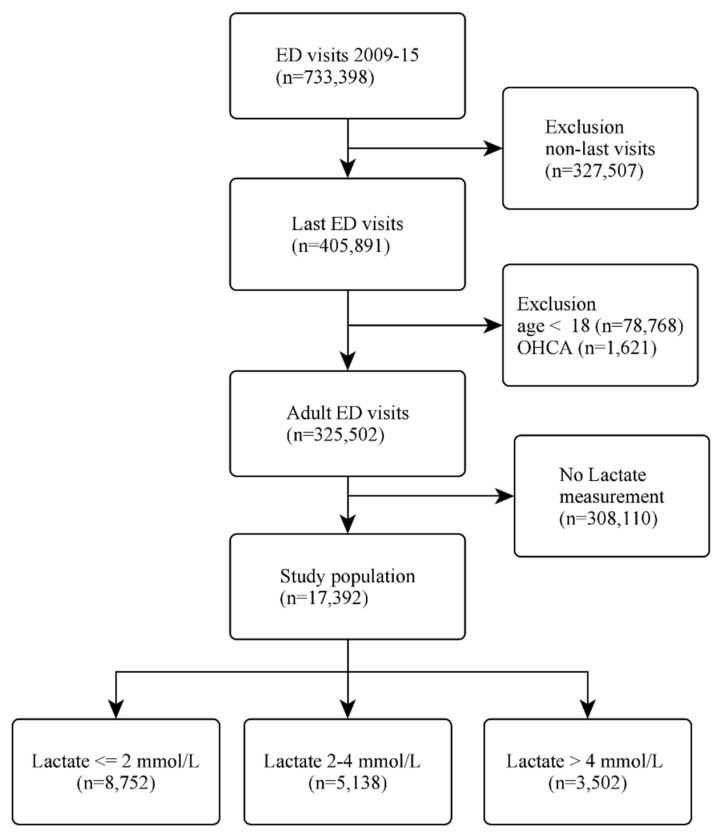
Flow diagram of the patient selection process. Abbreviations: ED, emergency department; OHCA, out-of-hospital cardiac arrest.

We extracted data from 733,398 ED visits over the 7-year period. If the patient presented to the ED more than once, we selected the last emergency visit to maximise the statistical power for the analysis of cardiac arrest. Patients were excluded if they were under 18 years of age, arrived at the ED with out-of-hospital cardiac arrest (OHCA), or if the serum lactate data were not available. The selection process is illustrated in Figure 1.

### 2.3. Variables

The patients’ demographic and time-stamped clinical information at triage were retrieved, including the chief complaint on presentation, mode of arrival, transfer status, vital signs (temperature, heart rate, systolic and diastolic blood pressure, respiratory rate, and oxygen saturation), and level of consciousness coded as the score of the Glasgow coma scale (GCS). The data extractors were hospital information technology engineers who were blinded to the study hypothesis. After the investigators’ meetings, the data underwent electronic cleaning, and invalid data were designated as missing values. Pain scores were evaluated using a numerical rating scale (NRS) of 0–10, where 0 indicated no pain, and 10 indicated the worst pain imaginable. We further categorised the NRS scores into no (0), mild (1–3), moderate (4–6), and severe (7–10) pain [28]. We also classified the level of consciousness as severe coma (GCS ≤ 8), moderate coma (GCS 9–12), and minor coma to normal status (GCS ≥ 13) [29]. Patients with special conditions, such as aphasia, tracheostomy, and endotracheal tube intubation, were classified as ‘others’ on the GCS evaluation. ED shifts were classified as day (07:00–14:59), evening (15:00–22:59), and night (23:00–06:59).

We extracted the scores of the five-level computerised Taiwan triage and acuity scale (TTAS), which contains information on 179 structured chief complaints. The chief complaints include OHCA, which was used to identify and exclude the patients that experienced OHCA. The TTAS classifies patients based on computerised algorithms: level 1, resuscitation; level 2, emergent; level 3, urgent; level 4, less urgent; and level 5, non-urgent. This acuity scale has been validated against hospitalisation and length of ED stay [30].

The initial serum lactate levels were divided into three groups: normal lactate level (≤2 mmol/L), moderately elevated lactate level (2 < lactate ≤ 4 mmol/L), and highly elevated lactate levels (>4 mmol/L) [31]. All lactate data used in the analysis were pre-IHCA measurements. Hourly lactate clearance was calculated using the following formula: Hourly lactate clearance = (initial lactate − follow-up lactate)/initial lactate × 100 (expressed as a percentage)/hours between initial and follow-up lactate measurements.

### 2.4. Outcome Measures

The primary outcome measure was IHCA in the ED (herein, ED-based IHCA), which was identified via the cardiopulmonary resuscitation (CPR) code in the patient record, thus indicating treatment of cardiac arrest. The secondary outcome was mortality in the ED. According to the consensus guidelines on reporting IHCA [32], the incidence of IHCA in the ED was calculated as the number of treated arrests as a proportion of the ED study population.

### 2.5. Statistical Analysis

Summary statistics are presented as proportions (with 95% confidence intervals (CIs)), means (with standard deviations (SDs)), or medians (with interquartile ranges (IQRs)). Bivariate associations were examined using Student’s *t*-test, the Mann–Whitney test, and chi-squared test, as appropriate. We used available-case analysis for the laboratory assessments, as the test results were not available for all patients.

We used generalised additive models to examine the potentially nonlinear relationship of lactataemia and lactate clearance with the risk of IHCA. We also used conditional plots to visualise the relationship between serum lactate and the impact of lactate clearance and identify potential inflection points after adjusting for age and sex. We used multivariable logistic regression to examine the independent association of lactate with ED-based IHCA. Variables that were strongly associated with the primary outcome measure on the bivariate analyses were considered for inclusion in the multivariable analysis. The discriminatory ability of the final models was evaluated using the area under the receiver operating curve (AUROC). To assess the impact of lactate clearance on the outcome, we also performed a subgroup analysis among patients who had an initially high lactate level (>2 mmol/L) and for whom a follow-up lactate measurement was available. Multivariable logistic regression was also performed to examine the independent association of lactate clearance with ED-based IHCA.

All odds ratios (ORs) and beta coefficients are presented with 95% CIs. All analyses were performed using Stata 16.0 software (StataCorp, College Station, TX, USA). All *p*-values were two-sided, and those less than 0.05 were considered to be statistically significant.

## 3. Results

Of the 733,398 ED visits during the 7-year study period, 405,891 unique patient visits were included in the study. After excluding children aged less than 18 years, patients with OHCA, and those who did not have a lactate measurement, 17,392 patients were included in the analysis. The outcome of the patient selection procedure is illustrated in Figure 1. There were 281 IHCA patients without a lactate measurement. Their triage to CPR time was similar to that in the 342 IHCA patients included in our study (7.3 h vs. 6.8 h, *p* = 0.53).

The baseline characteristics of the study population are shown in Table 1. The lactate levels were normal (lactate ≤ 2 mmol/L), moderately elevated (2 < lactate ≤ 4 mmol/L), and highly elevated (lactate > 4 mmol/L) in approximately 50%, 30%, and 20% of patients, respectively. Patients with moderately or highly elevated lactate levels tended to be older and predominantly male, compared with those in the normal lactate group. Patients with elevated lactate levels were more likely to arrive at the ED during weekends or at night. Patients in the elevated lactate group were more likely to arrive by ambulance, present with dyspnoea, and be triaged into higher levels (1 or 2), compared with the normal lactate group. Patients in the elevated lactate group were also more likely to present with impaired consciousness but were less likely to express pain. Compared with patients with normal lactate levels, patients with elevated lactate levels presented with higher heart and respiratory rates, but with slightly lower body temperature, oxygen saturation, and systolic blood pressure. Compared with patients with normal lactate levels, both the median time to IHCA and the median length of stay in the ED were shorter in patients with elevated lactate levels. The incidence of IHCA differed markedly across the three lactate groups (0.4% in the normal lactate group, 1.3% in the moderately elevated lactate group, and 6.9% in the highly elevated lactate group, *p* < 0.001). The rates of admission and ED mortality were significantly higher among patients with highly elevated lactate levels compared with patients with normal lactate levels.

Online Supplementary Table S1 summarizes the baseline characteristics of ED-based IHCA patients. The mean age was 67 years, and 60% of patients were male. Patients with IHCA were more likely to arrive at the ED in the morning and be triaged into higher levels (1 or 2). The most common ED discharge diagnoses included pneumonia (9.1%), followed by fever (5.3%), gastrointestinal bleeding (5%), chest pain (4.7%), and shock (4.4%). Additionally, 80.7% of patients received intubation, and 22.5% received cardioversion or defibrillation. The median time to IHCA was 6.8 h, and the median length of stay in ED was 8.4 h. Regarding ED disposition, 45% of the IHCA patients were admitted to the hospital, and 50.3% died in the ED.

The details of lactate measurements and other laboratory markers are shown in Table 2. Of note, the time from triage to the first measurement of lactate was shortest in the highly elevated lactate group. With regard to other laboratory markers, in general, patients in the highly elevated lactate group showed most abnormalities, including lower levels of haemoglobin, higher levels of white blood cells, more immature leukocytes, higher levels of creatinine, bilirubin, and potassium, lower pH and bicarbonate levels on arterial blood gas, and higher levels of troponin-I, C-reactive protein (CRP), and D-dimer.

**Figure 2:**
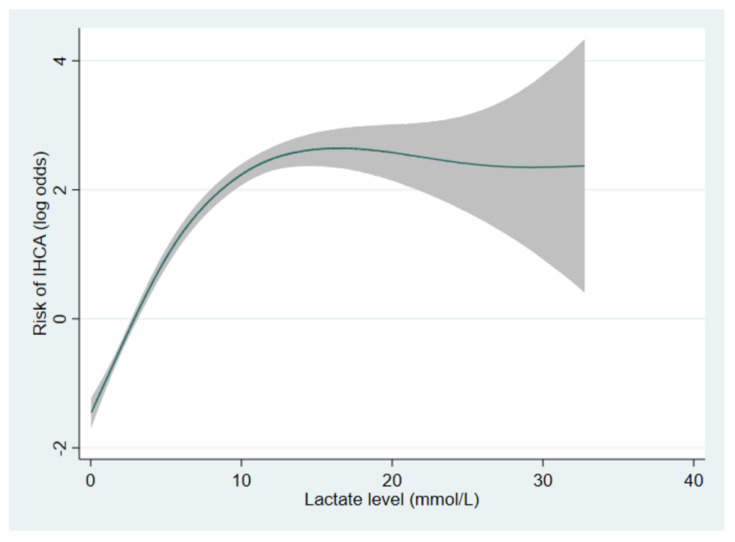
The generalised additive model showing the dose–response relationship between lactate levels and the risk of IHCA. The shaded area represents 95% confidence intervals. A log odds of zero represents the reference.

Multivariable analysis adjusting for 12 potential confounders revealed that the highly elevated lactate group was associated with an 18-fold higher risk of IHCA (adjusted OR, 18.0; 95% CI, 11.5–28.2), compared with the normal lactate group (Table 3). The model showed an excellent AUROC of 0.82. Figure 2 depicts the dose–response relationship between lactate levels and the risk of IHCA. The risk of IHCA increased with the elevation in lactate level in a positive linear manner when the lactate level was between approximately 0 and 10 mmol/L. The risk of IHCA appeared to have reached a diminishing return point at 10 mmol/L and then plateaued after 15 mmol/L. The confidence intervals beyond 15 mmol/L were quite wide due to few data points.

In this study, the median (interquartile range) of hourly lactate clearance was 2.3%/h (0.3–6.0%/h). Additionally, the generalised additive models (Online Supplementary Figure S1) showed a relatively negative, linear relationship between hourly lactate clearance and the risk of IHCA within most of the lactate clearance range. Based on these findings, we empirically defined normal lactate clearance as lactate reduction equal to or more than 2.5% per hour (≥2.5%/h), whereas a reduction of less than 2.5% per hour was defined as poor lactate clearance (<2.5%/h).

Online Supplementary Figure S2 is the profile plot showing the trend of lactate change in patients with follow-up lactate data. The peak lactate depends on the slope of lactate: with a rising slope, the peak lactate is the follow-up lactate, whereas with a downward slope, the peak lactate is the initial lactate value.

The subgroup analysis of patients with initial high lactate levels and follow-up measurement of lactate (*n* = 2481) is shown in Table 4. There were differences in baseline clinical characteristics by the lactate clearance group, as well as differences in outcomes. The poor-lactate clearance group (<2.5%/h) was associated with a higher incidence of IHCA (5% versus 1%, *p* < 0.001) compared with the normal clearance group. The poor lactate clearance group had a higher admission rate and a higher ED mortality rate. Multivariable analysis adjusting for potential confounders revealed that poor lactate clearance was associated with a 7.5-fold higher risk of IHCA (adjusted OR, 7.5; 95% CI, 3.7–15.1), compared with the normal clearance group (Table 5). The model AUROC was 0.79.

## 4. Discussion

This study found that, among 17,392 adult ED patients, the initial lactate level was associated with an increase in the risk of ED-based IHCA, and that a positive linear correlation existed between them when the lactate level was below approximately 10 mmol/L. Lactate clearance was also correlated with ED-based IHCA in a negative linear fashion, with a distribution-based cut-off of lactate clearance rate <2.5%/h identified.

Shapiro et al. reported that inpatient mortality rates increased with the elevation in lactate levels in ED patients with infection [19]. The 3-day inpatient mortality rates for the low (lactate < 2.5 mmol/L), medium (2.5 mmol/L ≤ lactate < 4 mmol/L), and high (lactate ≥ 4 mmol/L) groups were 1.5%, 4.5%, and 22.4%, respectively [19]. This study, however, did not include patients with conditions other than infection. A follow-up study included more clinical information and showed that lactate ≥4 mmol/L had seven times the odds of death in ED patients with infection after adjusting for other confounders [33]. Del Portal et al. reported that higher lactate values were correlated with greater mortality during hospitalisation at 30 days and 60 days in ED patients aged above 65 years [24]. Although that study included elderly individuals with a broad spectrum of conditions, the study results cannot be extrapolated to the younger ED population. In contrast to the three studies mentioned above, our study included adult ED patients with a variety of conditions, had the largest sample size to date, and focused on another clinically important outcome of ED-based IHCA.

Our study supports the notion that the initial lactate level provides useful predictive information about ED-based IHCA. Interestingly, the time to decision to order lactate was shortest among those who had the highest rate of IHCA, suggesting the appropriate indication and timely action of lactate measurement. Indeed, patients in the highly elevated group showed the shortest time to CPR, supporting the need for timely lactate measurement. Thus, the initial lactate level may serve as an early risk-stratification parameter in the ED. Higher levels of monitoring, if available, may be needed to prevent ED-based IHCA in patients with highly elevated lactate levels.

This study also reported that patients with poor lactate clearance were associated with a higher incidence of IHCA compared with those with normal clearance. Previous studies have suggested that repeated lactate measurement may be a more reliable prognostic predictor than the initial lactate value in sepsis [34,35]. Lower lactate clearance at 6 h [36], or even early (0–2 h) lactate clearance [37], is independently associated with a higher risk of mortality in patients with trauma. These studies used specific time intervals for repeated lactate follow-up measurements, which may not be feasible in the hectic ED environment. In this study, the lactate clearance was calculated with different time intervals, providing a more flexible and practical approach for serial lactate measurement in the ED. However, the cut-off value for lactate clearance varies. Promsin et al. reported that a lactate clearance of <2.5%/h was associated with higher 30-day mortality in ICU-treated patients with septic shock [38], a cut-off point that is concordant with our study results. Rising lactate levels may also indicate some limits to ED treatments. For example, emergent endoscopy for gastrointestinal bleeding patients may not be readily available at midnight. These treatment limitations may lead to rising lactate levels and poor lactate clearance, leading to IHCA.

## 5. Limitations

The present study was conducted at a single tertiary medical centre, which hinders generalisability to other community hospitals, particularly the availability of follow-up lactate measurements. Second, this was a retrospective study where physicians ordered lactate measurements at their discretion, resulting in a smaller and sicker population with greater hyperlactataemia. Thus, the distribution of lactate levels may be shifted toward the higher end; however, the association of lactataemia with outcomes should still hold true. Third, the follow-up lactate measurements were not available for all patients with initially elevated lactate levels. As such, the cut-off of 2.5%/h identified in our study should be validated in future studies. Moreover, many patients may have also been transferred to the ICU before their condition worsened, indicating that follow-up lactate levels may have increased following ICU admission and may have affected our data. Finally, as with any observational studies, our findings may be subject to unmeasured confounders, such as comorbidities (not well documented in the ED). 

## 6. Conclusions

In conclusion, in this ED study of 17,392 adult patients, elevated lactate levels were strongly associated with a higher risk of IHCA in the ED; the two showed a positive correlation when the lactate level was below approximately 10 mg/dL. Lactate clearance <2.5%/h was associated with a higher risk of ED-based IHCA. Clinicians should consider liberal lactate measurements in patients at a higher risk of IHCA and follow up on those with abnormal lactate levels. Future research is warranted to determine if a more liberal sampling of lactate and appropriate interventions could reduce ED-based IHCA and associated mortality.

## Figures and Tables

**Table 1:** Baseline patient characteristics by the lactate group.

Variable	Lactate ≤ 2 mmol/L (*n* = 8752)	2 < Lactate ≤ 4 mmol/L (*n* = 5138)	Lactate > 4 mmol/L (*n* = 3502)	*p* Value
Age, mean (SD), yr	62.2 (19.6)	66.1 (17.9)	65.9 (17.2)	<0.001
Female sex, *n* (%)	4277 (48.9)	2136 (41.6)	1369 (39.1)	<0.001
Weekend, *n* (%)	2239 (25.6)	1411 (27.5)	948 (27.1)	0.033
Time of presentation, *n* (%)				<0.001
7:00 a.m. to 2:59 p.m.	3688 (42.1)	2230 (43.4)	1440 (41.1)	
3:00 p.m. to 10:59 p.m.	3744 (42.8)	2059 (40.1)	1389 (39.7)	
11:00 p.m. to 6:59 a.m.	1320 (15.1)	849 (16.5)	673 (19.2)	
Arrival by ambulance, *n* (%)	1874 (21.4)	1587 (30.9)	1425 (40.7)	<0.001
Presenting chief complaint, *n* (%)				<0.001
Abdominal pain	2005 (22.9)	727 (14.1)	224 (6.4)	
Fever	1122 (12.8)	593 (11.5)	259 (7.4)	
Dizziness	213 (2.4)	119 (2.3)	42 (1.2)	
Dyspnea	1291 (14.8)	1037 (20.2)	962 (27.5)	
Chest pain	194 (2.2)	114 (2.2)	71 (2.0)	
Other	3927 (44.9)	2548 (49.6)	1944 (55.5)	
Triage level, *n* (%)				<0.001
1	1151 (13.2)	1326 (25.8)	1620 (46.3)	
2	3312 (37.8)	2105 (41.0)	1244 (35.5)	
3	4104 (46.9)	1647 (32.1)	614 (17.5)	
4	166 (1.9)	53 (1.0)	21 (0.6)	
5	19 (0.2)	7 (0.1)	3 (0.1)	
GCS, *n* (%)				<0.001
13–15	7343 (83.9)	3881 (75.5)	2266 (64.7)	
9–12	647 (7.4)	560 (10.9)	448 (12.8)	
3–8	383 (4.4)	405 (7.9)	500 (14.3)	
Other (A, E, T)	379 (4.3)	292 (5.7)	288 (8.2)	
Pain score, *n* (%)				<0.001
Severe (7–10)	1624 (18.7)	722 (14.2)	322 (9.5)	
Moderate (4–6)	1271 (14.7)	461 (9.1)	156 (4.6)	
Mild (1–3)	112 (1.3)	39 (0.8)	18 (0.5)	
No pain (0)	5662 (65.3)	3849 (75.9)	2907 (85.4)	
Body temperature, mean (SD), °C	37.2 (1.1)	37.2 (1.2)	37.0 (1.4)	<0.001
Heart rate, mean (SD), beats per min	93.5 (22.0)	100.5 (25.1)	108.7 (27.4)	<0.001
Respiratory rate, mean (SD), breaths per min	19.5 (3.2)	20.4 (3.9)	21.8 (5.1)	<0.001
Oxygen saturation, median (IQR), %	97.0 (95.0–98.0)	96.0 (94.0–98.0)	96.0 (92.0–98.0)	<0.001
Systolic blood pressure, mean (SD), mmHg	131.7 (31.4)	127.2 (35.2)	118.1 (37.4)	<0.001
Triage to CPR time, median (IQR), hours	21.7 (7.0–47.2)	19.6 (3.7–48.6)	5.3 (2.7–15.2)	<0.001
ED length of stay, median (IQR), hours	24.4 (6.0–66.0)	25.5 (6.2–67.9)	15.5 (4.3–48.6)	<0.001
IHCA, *n* (%)	35 (0.4)	67 (1.3)	240 (6.9)	<0.001
Discharge status, *n* (%)				<0.001
Discharge	3123 (35.7)	1209 (23.5)	297 (8.5)	
Admission	4925 (56.3)	3387 (65.9)	2477 (70.7)	
Death	206 (2.4)	265 (5.2)	557 (15.9)	
Other	497 (5.7)	277 (5.4)	171 (4.9)	

Abbreviations: GCS = Glasgow coma scale; GCS-A = aphasia; GCS-E = endotracheal tube; GCS-T = tracheostomy; SD = standard deviation; IQR = interquartile range; ED = emergency department; IHCA = in-hospital cardiac arrest.

**Table 2:** Laboratory markers by the lactate group.

Variable	Lactate ≤2 mmol/L (*n* = 8752)	2 < Lactate ≤4 mmol/L (*n* = 5138)	Lactate > 4 mmol/L (*n* = 3502)	*p* Value
Lactic acid, median (IQR), mmol/L	1.3 (1.0–1.6)	2.7 (2.3–3.2)	6.5 (5.0–9.8)	<0.001
Triage to first lactate measurement, median (IQR), hours	1.2 (0.7–2.8)	1.0 (0.6–2.0)	1.0 (0.5–2.1)	<0.001
Haemoglobin, median (IQR), g/dL (*n* = 16,916)	12.1 (10.1–13.8)	12.2 (10.3–14.2)	11.7 (9.5–14.0)	<0.001
Platelet count, median (IQR), K/μL (*n* = 16,916)	208.0 (153.0–267.0)	198.0 (141.0–261.0)	184.0 (114.0–264.0)	0.218
White blood cell count, median (IQR), K/μL (*n* = 16,916)	9.4 (6.8–13.1)	10.8 (7.5–15.1)	12.0 (7.7–17.7)	<0.001
Band form of leukocyte, median (IQR), % (*n* = 16,617)	0.0 (0.0–0.0)	0.0 (0.0–0.0)	0.0 (0.0–5.7)	<0.001
Creatinine, median (IQR), mg/dL (*n* = 17,175)	0.9 (0.7–1.5)	1.1 (0.8–1.7)	1.5 (1.0–2.4)	<0.001
Total bilirubin, median (IQR), mg/dL (*n* = 9255)	0.8 (0.5–1.2)	0.9 (0.6–1.7)	1.3 (0.7–3.1)	<0.001
Potassium, median (IQR), mmol/L (*n* = 17,118)	4.1 (3.8–4.7)	4.3 (3.8–4.9)	4.5 (3.8–5.3)	<0.001
pH, median (IQR) (*n* = 9177)	7.4 (7.4–7.4)	7.4 (7.4–7.4)	7.4 (7.3–7.4)	<0.001
HCO3-, median (IQR), mEq/L (*n* = 9176)	23.7 (20.8–26.5)	23.1 (20.0–26.2)	19.8 (15.9–23.4)	<0.001
Troponin I, median (IQR), ng/mL (*n* = 9313)	0.0 (0.0–0.1)	0.0 (0.0–0.1)	0.1 (0.0–0.2)	<0.001
CRP, median (IQR), mg/dL (*n* = 3577)	4.4 (0.8–11.5)	5.4 (1.1–13.5)	6.3 (1.6–13.7)	<0.001
D-dimer, median (IQR), μg/mL (*n* = 1899)	2.0 (0.7–4.4)	2.5 (0.9–6.1)	5.1 (2.2–12.2)	<0.001

Abbreviations: IQR = interquartile range; CRP = C-reactive protein.

**Table 3:** Multivariable model of factors associated with emergency department-based in-hospital cardiac arrest.

Variable	Adjusted Odds Ratio	95% Confidence Interval	*p* Value
Lactate, mmol/L			
≤2 (reference)	1.0		
2–4	3.1	1.9–5.1	<0.001
>4	18.0	11.0–28.2	<0.001
Age, per 10-year increase	1.1	1.0–1.2	0.01
Chief complaint			
Abdominal pain (vs. other)	0.4	0.2–0.8	0.01
Chest pain (vs. other)	2.0	1.0–4.0	0.04
Day (7 a.m.–3 p.m.) vs. evening shift	1.5	1.1–2.1	0.01

The model also adjusted for the following variables that were not statistically significant (not shown in Table): weekend, arrival by ambulance, pain score, Glasgow coma scale, body temperature, heart rate, respiratory rate, oxygen saturation, and systolic blood pressure.

**Table 4:** Baseline clinical characteristics and outcomes by lactate clearance.

Variable		Lactate Clearance < 2.5%/h (*n* = 1289)	Lactate Clearance ≥ 2.5%/h (*n* = 1192)	*p*-Value
Age, mean (SD), yr		69.1 (15.9)	66.8 (17.4)	0.002
Female sex, *n* (%)		513 (39.8)	481 (40.4)	0.78
Weekend, *n* (%)		353 (27.4)	343 (28.8)	0.44
Time of presentation, *n* (%)				0.90
	7:00 a.m. to 2:59 p.m.	542 (42.1)	492 (41.3)	
	3:00 p.m. to 10:59 p.m.	524 (40.7)	495 (41.5)	
	11:00 p.m. to 6:59 a.m.	223 (17.3)	205 (17.2)	
Presenting chief complaint, *n* (%)				<0.001
	Dyspnea	299 (45.8)	249 (44.5)	
	Chest pain	12 (1.8)	32 (5.7)	
	Abdominal pain	139 (21.3)	133 (23.8)	
	Dizziness	23 (3.5)	26 (4.6)	
	Fever	180 (27.6)	120 (21.4)	
Arrival by ambulance, *n* (%)		432 (33.5)	393 (33.0)	0.77
Triage level, *n* (%)				<0.001
	1	342 (26.5)	412 (34.6)	
	2	527 (40.9)	466 (39.1)	
	3	408 (31.7)	304 (25.5)	
	4	11 (0.9)	9 (0.8)	
	5	1 (0.1)	1 (0.1)	
Pain score, *n* (%)				0.02
	Severe (7–10)	141 (11.2)	144 (12.3)	
	Moderate (4–6)	109 (8.6)	78 (6.7)	
	Mild (1–3)	2 (0.2)	10 (0.9)	
	No pain (0)	1014 (80.1)	942 (80.2)	
GCS, *n* (%)				0.03
	13–15	935 (72.6)	893 (74.9)	
	9–12	185 (14.4)	149 (12.5)	
	3–8	103 (8.0)	112 (9.4)	
	Other (A, E, T)	66 (5.1)	38 (3.2)	
Body Temperature, mean (SD), °C		37.3 (1.3)	37.2 (1.3)	0.32
Heart rate, mean (SD), beats per min		106.6 (25.3)	106.2 (27.4)	0.005
Systolic blood pressure, mean (SD), mmHg		119.7 (32.8)	116.5 (36.5)	<0.001
Respiratory rate, mean (SD), breaths per min		20.8 (4.0)	21.0 (4.4)	<0.001
Oxygen saturation, median (IQR), %		96 (94.0–98.0)	96 (94.0–98.0)	0.45
ED length of stay, median (IQR), h		62.1 (25.5–110.4)	40.6 (16.4–75.3)	<0.001
IHCA, *n* (%)		68 (5.3)	9 (0.8)	<0.001
Discharge status, *n* (%)				<0.001
	Discharge	91 (7.1)	250 (21.0)	
	Admission	1011 (78.4)	824 (69.1)	
	Death	149 (11.6)	69 (5.8)	
	Other	38 (3.0)	49 (4.1)	

Abbreviations: GCS = Glasgow coma scale; GCS-A = aphasia; GCS-E = endotracheal tube; GCS-T = tracheostomy; SD = standard deviation; IQR = interquartile range; ED = emergency department; IHCA = in-hospital cardiac arrest.

**Table 5:** Multivariable model of factors associated with emergency department-based in-hospital cardiac arrest among the subset of calculable lactate clearance.

Variable	Adjusted Odds Ratio	95% Confidence Interval	*p* Value
Lactate clearance, %/h			
≥2.5 (reference)	1.0		
<2.5	7.5	3.7–15.1	<0.001
Arrival by ambulance	1.7	1.04–2.7	0.04

The model also adjusted for the following variables that were not statistically significant (not shown in Table): age, sex.

## Data Availability

The datasets used and/or analysed during the current study are available from the corresponding author on reasonable request.

## Supplementary Materials

The following are available online at https://www.mdpi.com/article/10.3390/jcm11020403/s1, Supplement Table S1: Baseline clinical characteristics of emergency department patients with in-hospital cardiac arrest, Supplement Figure S1: Relationship between hourly lactate clearance and the risk of IHCA, Supplement Figure S2: Profile plot showing the trend of change in lactate levels in patients with follow-up lactate data. The red line indicates the change in average values.
