# Supplementary material for: Serum Lactate for Predicting Cardiac Arrest in the Emergency Department
Source: J Clin Med. 2022 Jan 13;11(2):403. doi: 10.3390/jcm11020403 (PMC8778773; doi:10.3390/jcm11020403)
Supplement: Supplementary file 1 [file jcm-11-00403-s001.zip › jcm-1486935-supplementary.pdf]

Supplementary Table S1. Baseline clinical characteristics of emergency department patients with in-hospital cardiac arrest (N=342).

**Variables**	
Age, year, mean (SD)	67.6 (16.3)
Female sex, n (%)	138 (40.4)
Season, n (%)	
Spring (March–May)	82 (24.0)
Summer (June–August)	78 (22.8)
Fall (September–November)	89 (26.0)
Winter (December–February)	93 (27.2)
Presenting time, n (%)	
7:00 am to 2:59 pm	156 (45.6)
3:00 pm to 10:59 pm	123 (36.0)
11:00 pm to 6:59 am	63 (18.4)
Common discharge diagnosis, n (%)	
Pneumonia	31 (9.1)
Fever	18 (5.3)
Gastrointestinal bleeding	17 (5.0)
Chest pain	16 (4.7)
Shock	15 (4.4)
Triage level, n (%)	
1	151 (44.2)
2	113 (33.0)
3	71 (20.8)
4	6 (1.8)
5	1 (0.3)
Vital sign at triage	
Systolic blood pressure, mmHg, mean (SD)	114.6 (35.6)
Heart rate, beats per min, mean (SD)	99.4 (27.8)
Body temperature, °C, mean (SD)	36.9 (1.4)
Respiratory rate, breaths per min, mean (SD)	21.4 (4.6)
Oxygen saturation, %, median (IQR)	96 (92–98)
Time to IHCA, h, median (IQR)	6.8 (3.2–26.1)
Intubated, n (%)	276 (80.7)

Cardioversion or defibrillation, n (%)	77 (22.5)
Hospital admission, n (%)	154 (45.0)
ED mortality, n (%)	172 (50.3)
ED/EDOU length of stay, median (IQR), h	8.4 (3.6–27.2)

Abbreviations: SD, standard deviation; IQR, interquartile range; IHCA, in-hospital cardiac arrest; ED, emergency department; EDOU, emergency department observation unit.

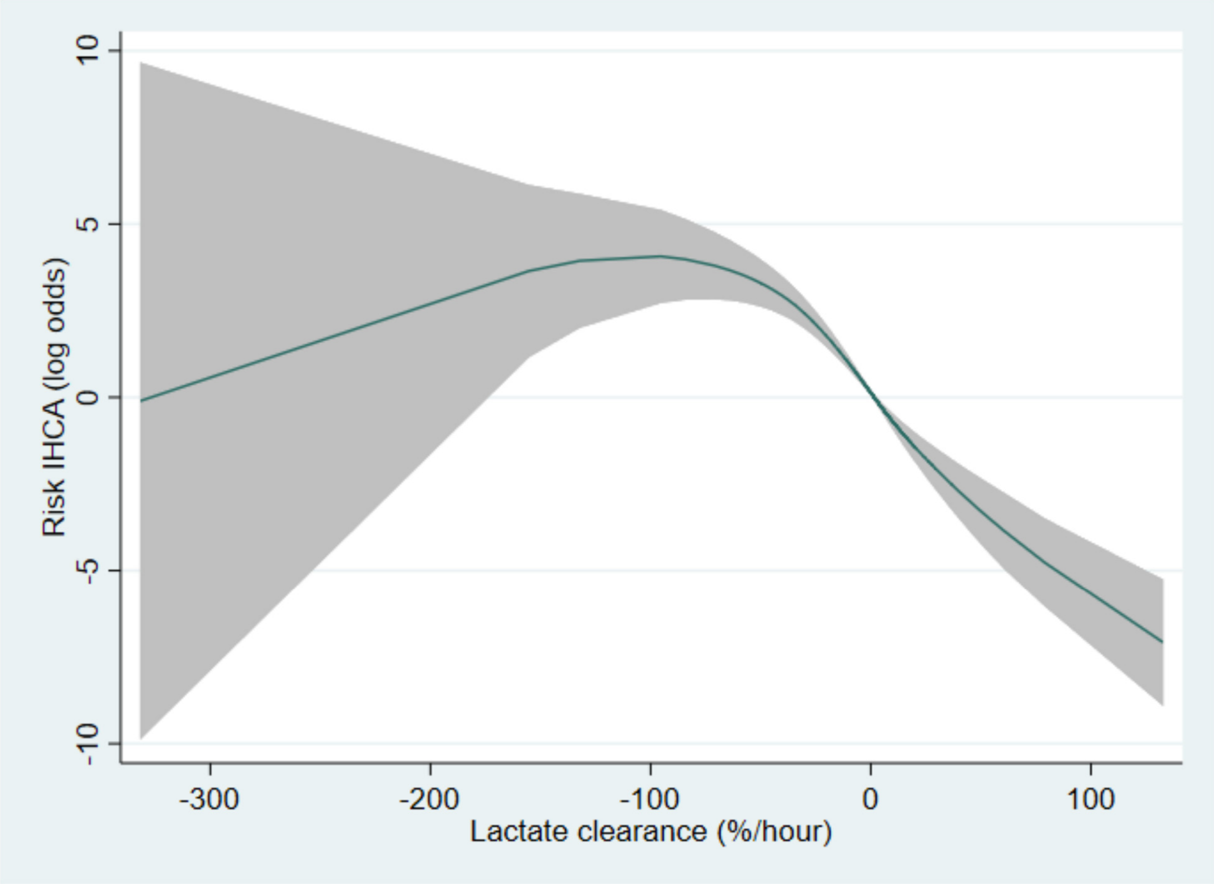

Figure S1. Relationship between hourly lactate clearance and the risk of IHCA.

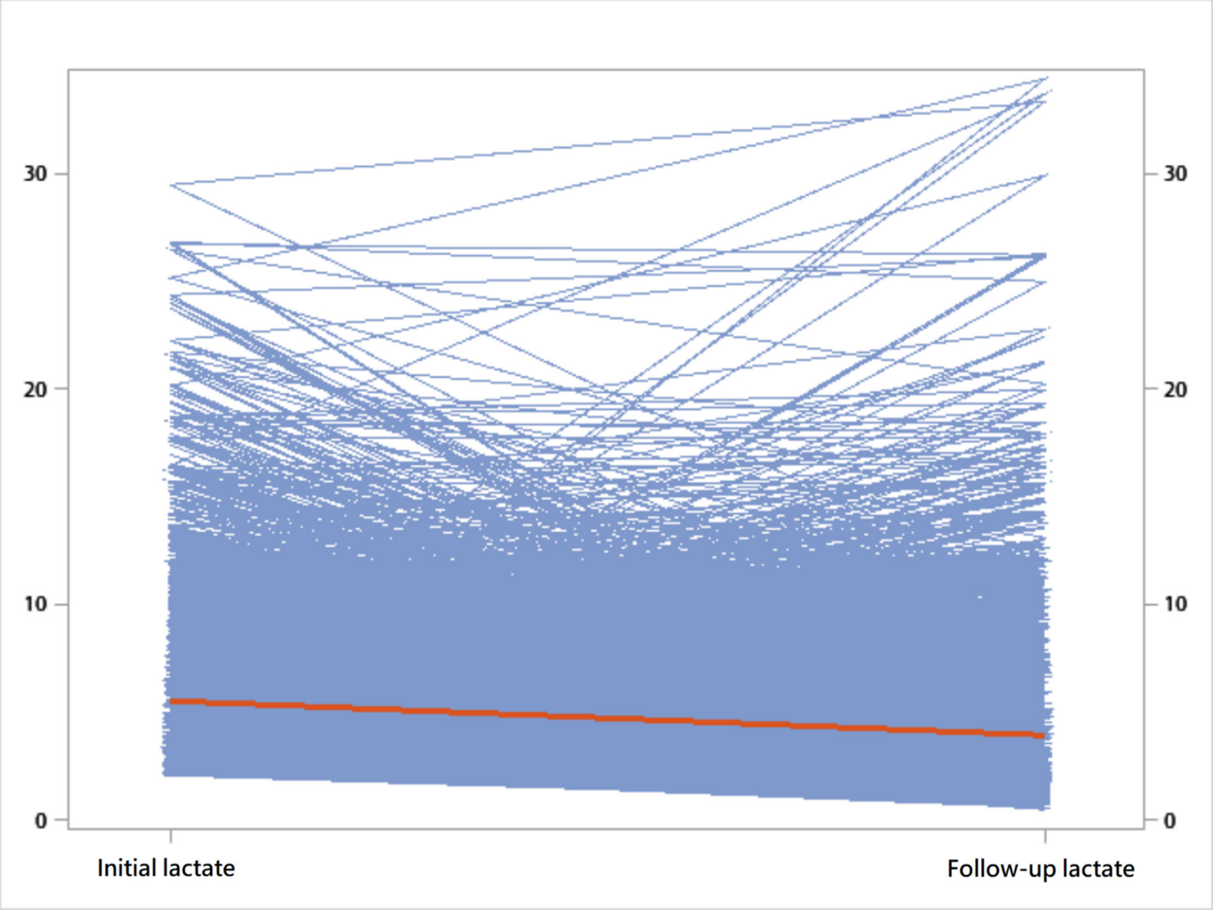

Figure S2. Profile plot showing the trend of change in lactate levels in patients with follow-up lactate data. The red line indicates the change in average values.
